# Effect of Tecar Therapy in the Treatment of Skin Flaccidity and Localized Abdominal Fat—Clinical and Controlled Study

**DOI:** 10.1155/drp/5531539

**Published:** 2026-02-23

**Authors:** Stephani de Almeida, Isabella Benatti Santiago, Cintia Cristina Santi Martignago, Michele Akemi Nishioka, Patricia Brassolatti, Jéssica Helena Franco Dorigatti, Fabiele Chieregato, Ana Carolina Araruna Alves, Stephany Luanna Queiroga Farias, Patricia Froes Meyer, José Ricardo de Souza

**Affiliations:** ^1^ Postgraduate Degree in Human Movement Science and Rehabilitation, Federal University of São Paulo (UNIFESP), Santos, São Paulo, Brazil, unifesp.br; ^2^ Biomedicine Graduated, São Francisco University-USF, Bragança Paulista, São Paulo, Brazil; ^3^ Department of Biosciences, Federal University of São Paulo (UNIFESP), Santos, Brazil, unifesp.br; ^4^ Postgraduate Degree in Plastic Surgery From the Federal University of São Paulo (UNIFESP), São Paulo, São Paulo, Brazil; ^5^ Postgraduate Degree in Biotecnology From the Federal University of São Carlos (UFSCAR), São Carlos, São Paulo, Brazil; ^6^ Postgraduate Degree in Biomedical Engineering, University of Brazil, São Paulo, Brazil; ^7^ Postgraduate Degree in Rehabilitation Sciences, Universidade Nove de Julho, São Paulo, Brazil, uninove.br; ^8^ Department of Physiotherapy, International Research Group–IRG, Natal, Rio Grande do Norte, Brazil; ^9^ Department of Physiotherapy, Federal University of Rio Grande Do Norte (UFRN), Natal, Rio Grande do Norte, Brazil, ufrn.br; ^10^ Department of Research, Development and Innovation, Industria Brasileira de Equipamentos Eletromédicos (IBRAMED), Amparo, São Paulo, Brazil

**Keywords:** localized fat, tecar therapy, tissue histology, tissue sagging

## Abstract

It is known that when an electrical stimulus is greater than 10 kHz, our body converts this electrical energy into thermal energy, and due to this fact, one of the therapies that has been showing good results is therapeutic, capacitive, and resistive energy transfer (tecar therapy), but there is still little evidence about its action on area of aesthetics. Therefore, the objective of this study was to evaluate the safety and efficacy of tecar therapy for the reduction of localized fat and improvement of skin flaccidity in the abdomen region. A controlled clinical trial was carried out with 48 women equally divided into 2 distinct groups, with (G‐1) received treatment with tecar therapy and (G‐2) considered the control. Ten treatment sessions were performed once a week. The volunteers were evaluated at three different times using anthropometric data, adipometry, and ultrasound (US), in addition to histological analysis of the adipose tissue performed on one of the volunteers. In the results of the adipometry and US, a decrease was observed both in the comparison between the groups and between the baseline and the end of the treatment for the supra and infra umbilical region. In the histological analysis, it was observed that the G1 showed positive markers for moderate chronic inflammation, indicating degeneration of the adipose tissue with a large number of fibroblasts and newly formed blood vessels in the integumentary tissue. With this, it was possible to conclude that tecar therapy proved to be a safe and effective resource in the treatment of sagging skin and reduction of superficial adipose tissue.

**Trial Registration:** ClinicalTrials.gov identifier: NCT05020054

## 1. Introduction

Physical therapies based on electrical or electromagnetic stimulation are used for rehabilitation and esthetic treatments. In this context, electrical stimulation therapies have been combined to stimulate heat production; as in diathermy, one of these technologies being therapeutic capacitive and resistive energy transfer (tecar therapy). This is the latest therapeutic modality in the range of electrophysical agents. tTe therapy consists of the use of high‐frequency electrical currents that favor the production of thermal and nonthermal effects in tissues without causing muscle contraction [1, 2].

Classified as deep thermotherapy, the tecar therapy initially operated only within the radiofrequency (RF) range that comprised 300 kHz–1.2 MHz; however, nowadays, with technological advances, it already comprises other ranges, making it possible to use frequencies from 0.5 to 4 MHz, allowing its performance in different anatomical structures such as integumentary tissue, superficial subcutaneous adipose tissue, and muscle tissue, the latter being one of the first scientific evidences of this type of therapeutic approach [1–3].

The physiological effects of diathermy or athermia are provided to the tissues through capacitive and resistive electrodes, but initially the therapy was performed only with capacitive electrodes, being called tecar. In 1985, this therapy became known as tecar therapy—Capacitive and Resistive Electrical Transfer, where in addition to the use of capacitive electrodes, resistive electrodes were also added, thus initiating a technological reformulation that allowed the use of different frequencies with high powers [1–3]. As a result, therapy has been used in rehabilitation and sports medicine to treat injuries to various structures such as muscles, bones, ligaments, and tendons with satisfactory results, and more recently, it has been used in the treatment of esthetic conditions such as wrinkles and sagging skin, as well as, in the treatment of cellulite and localized fat [4–6].

Tecar therapy is considered a RF modality about its physical principles, with established differences in the forms of diathermy generation, which in tecar therapy, is by electric current, and in RF, is by the formation of the electromagnetic field, and therefore, it is believed that the physiological effects, indications for use and contraindications of RF can be used in tecar therapy [5–7]. Thus, tecar therapy can be an innovative resource and present beneficial effects in the treatment of disorders involving the integumentary and superficial subcutaneous tissue [1–3]. Therefore, the present study aimed to evaluate the mechanism of action through a clinical and histological analysis of skin sagging and reduction of superficial adipose tissue, that is, fat located in the abdomen region, through a randomized and controlled clinical study.

## 2. Materials and Methods

### 2.1. Ethical Considerations

This is a multicenter randomized controlled clinical trial (RTC) with an experimental arm approved by the institutional ethics committee of Universidade Federal do Rio Grande do Norte— UFRN under opinion 4.062.975. The research was carried out in partnership between the research centers —Center for Studies and Advanced Training, located in the city of Amparo‐ SP, Brazil, IBRAMED—Brazilian medical device company, Amparo/SP, Brazil and the International Research Group (IRG), located in Natal‐RN, Brazil.

### 2.2. Study Population

The inclusion criteria included women aged between 30 and 45 years, with a body mass index (BMI) considered normal or overweight (18.5–29.9 kg/cm^2^), sedentary, with complaints of localized fat above 1.5 cm and abdominal skin sagging, they could not be under other esthetic treatments for the same purpose, and the ability to understand and preserved local sensitivity was necessary. Exclusion criteria for the study included women who smoked, were pregnant, had metabolic disorders, a history of deep vein thrombosis (DVT), neoplasms, had skin lesions over the treatment area, and who had an implanted electronic device such as a cardiac pacemaker and implant metal in the treatment area.

### 2.3. Data Collect

Anthropometric data on weight (kg), height (m^2^), and BMI (kg/height m^2^) were collected; the assessment of abdominal circumference was performed with a measuring tape (Fiber®), where the abdomen was subdivided into supraumbilical abdomen, umbilical line and infraumbilical abdomen. To measure the thickness of the superficial subcutaneous adipose tissue, adipometry (CESCORF®) was performed, where the folds were collected approximately 5 cm to the right of the umbilicus, parallel to the longitudinal axis. The thickness of the superficial subcutaneous tissue was also measured using a diagnostic ultrasound examination with a linear transducer (frequency 6–18 MHZ) (MYLAB™ 25 GOLD, ESAOTE, Italy) and VPAN software (ESAOTE, Italy). The marking of the evaluation point was 5 cm to the right of the umbilicus with the participant standing upright and for the measurement the participant was positioned in dorsal decubitus. The registration of the photos was carried out in orthostatism in the lateral view (right and left). The camera was positioned on a support tripod at a height of 66 cm from the ground, and it was placed at 55 cm from the participant, for better visualization and standardization of the photos. Data collection was carried out in three different moments, an initial assessment, and reassessments after 5 sessions and after 7 days of the completion of the 10 treatment sessions.

### 2.4. Experimental Protocol

A total of 48 patients participated in the study, but only 38 completed all the evaluation stages. The participants were divided into two distinct groups, with G‐1 being the group treated with thecartherapy and G‐2 considered as the control, in which they received the treatment with the equipment turned off.

### 2.5. Treatment Procedures

All study participants received the same treatment protocol, once a week in the abdomen and flanks region, totaling 10 treatment sessions, with the parameters described in Table 1. The treatment areas were cleaned with antiseptic soap, and a conductive cream was used to apply the therapy (Nèartek‐Essencial Cosmetic Company LTDA). Therapy was applied with dynamic movements, and temperature assessment was performed throughout the session. The total treatment time for the therapy on the abdomen and flanks was 60 min, being this time subdivided into areas of application mentioned above. In order to provide a homogeneous heating, the abdomen was divided into left and right (300 cm^2^ on each side) being applied for 10 capacitive minutes followed by equal resistive time, as for each flank, it was applied for 5 capacitive minutes followed by 5 resistive minutes. Both groups received the therapy or its simulation with the tecar therapy equipment, Nèartek®—IBRAMED—Brazilian medical device company.

**TABLE 1:** Parameters used in the application of tecar therapy.

Parameters of tecar therapy		
Treatment area (cm^2^)	300	
Frequency (MHz)	1	
Modality	Capacitive	Resistive
Power (W)	100	110
Applicator size (mm)	60	60
Treatment time (min)	10	10
Temperature °C	42	42

### 2.6. Statistical Analysis

Descriptive and inferential statistics of the data were performed using the Statistical Package for the Social Sciences—Version 21.0 (SPSS 21.0 program). Their normality was observed using the Kolmogorov–Smirnov (KS) test and the mixed ANOVA test was used to compare the groups. A significance level of 5% (*p* < 0.05) was adopted.

## 3. Results

Forty‐eight women participated in the study, 24 in each group; however, in the reassessment after the completion of the 10 therapy sessions, only 38 women will attend, resulting in a 12% decrease in the sample in Table 2. It is worth noting that no serious adverse events such as edema, hematoma, and burns were reported during the entire treatment period. Local erythema after the procedure was transient and did not persist for more than 30 min.

**TABLE 2:** Description of the sample with mean and standard deviation of anthropometric data.

Group	Baseline (*N* = 24)	5 sessions (*N* = 24)	10 sessions (*N* = 38)
Anthropometric data			
G‐1 Treatment			
Participants number	24	24	18
Age	39.33 ± 6.42	39.33 ± 6.42	39.33 ± 6.42
Body weight (kg)	70.33 ± 1.88	70.73 ± 1.93	70.57 ± 1.99
Height (cm)	1.6 ± 0.01	1.6 ± 0.1	1.6 ± 0.1
IMC (kg/cm^2^)	27.19 ± 0.53	27.11 ± 0.53	27.27 ± 0.62
G‐2 Control			
Participants number	24	24	20
Age	39.13 ± 6.12	39.13 ± 6.12	39.13 ± 6.12
Body weight (kg)	70.51 ± 2.20	68.08 ± 2.28	70.25 ± 2.61
Height (cm)	1.64 ± 0.1	1.64 ± 0.1	1.64 ± 0.1
IMC	25.91 ± 0.57	25.2 ± 0.59	24.75 ± 1.65

In the analysis of abdominal circumference, the variables of umbilical and infraumbilical circumference of G‐1 showed a significant reduction of *p* < 0.04 (umbilical) and *p* < 0.02 (infraumbilical) when compared to baseline and posttreatment with 10 sessions of tecar therapy in relation to placebo G‐2. There was no significant difference when comparing groups G‐1 and G‐2 in the 5‐session reassessment. The analysis of the adipometry data did not show a significant difference in any of the evaluated measurements and session interval, when the groups were compared.

**FIGURE 1:**
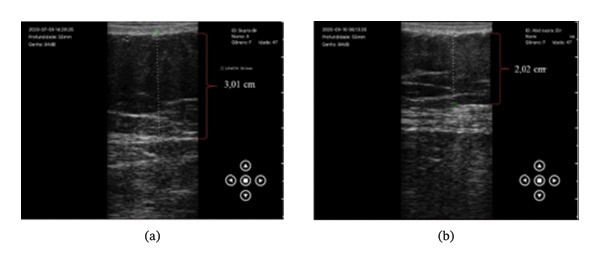
Ultrasonographic image of the abdomen of one of the participants in the G‐1 research, demonstrating the reduction of superficial adipose tissue, after the first sessions of tecar therapy.

In the analysis of the adipose tissue reduction of both groups, there was a significant difference in the comparisons, and the G‐1 showed a significant difference between the baseline and the end of the 10 treatment sessions in the measurements of the supraumbilical and infraumbilical regions of *p* = 0.002 and *p* = 0.001 when compared to G‐2. In the evaluations of the umbilical region and in relation to the interval of 5 treatment sessions, no statistical difference was found. Figure 1 demonstrates the reduction of adipose tissue in the supraumbilical abdominal region.

### 3.1. Patient Satisfaction

Results of the patient satisfaction questionnaire regarding improvement in skin sagging showed that 61% of participants reported improvement in skin sagging in the control group compared to 40.9% reported by participants in the treated group. When asked about the type of treatment, in G‐1, 68.18% of the participants reported it as excellent, 13.63% as very good, 13.63% as good, and 4.5 as poor treatment. In the G‐2, 55.5% reported it as excellent, 33.3% as very good, 5.5% as good, and 5.5% as poor. The global esthetic improvement scale revealed that 100% of the participants in G‐1 reported an excellent sagging result after treatment. As for the control group, differences were found in relation to this report in which 55.5% of the participants reported an excellent result after treatment, 16.6% as a marked improvement in the appearance of the initial condition, 16.6% as an obvious improvement in the appearance of the baseline condition and 11.11% being the appearance essentially the same as the baseline condition. Regarding satisfaction at the end of treatment, G‐1 participants demonstrated 100% satisfaction compared to 95% satisfaction reported by G‐2.

### 3.2. Histological Analysis

**FIGURE 2:**
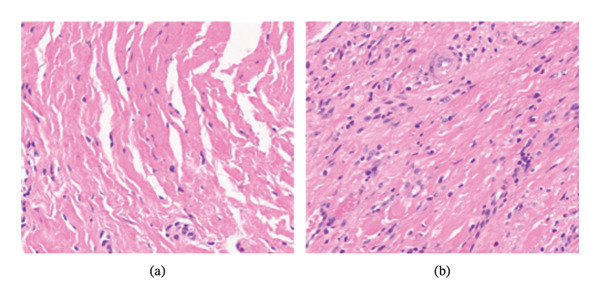
Photomicrographs of the intervention and placebo groups. (a) In the superficial dermis, the placebo group presented collagen tissue with little cellularity and absence of inflammatory cells, with aspects of normality. (b) The intervention group showed a large number of fibroblasts with collagen deposition and presence of inflammatory cells and neoangiogênese. The staining method used was Hematoxylin and Eosin (HE), and all photographs were taken at 20 × magnification field.

The result of the morphological analysis of the tegument tissue with Hematoxylin and Eosin (HE) showed that in the sample taken from the region did not undergo treatment. In the control group, there was collagen tissue with little cellularity and absence of inflammatory cells. The samples from the treated group, collected from the region that received the treatment, showed a large number of fibroblasts with collagen deposition and inflammatory cells, as seen in Figure 2.

**FIGURE 3:**
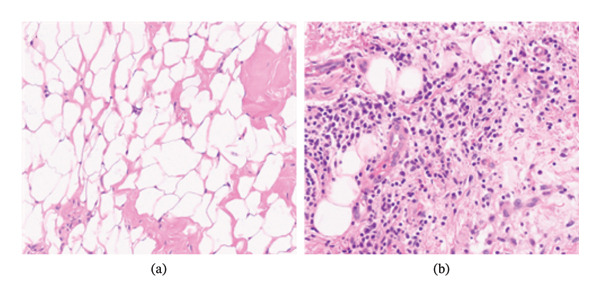
Photomicrographs of the intervention and placebo groups. (a) In superficial adipose tissue, the placebo group had adipose tissue with standard‐sized adipocytes and absence of inflammation. (b) The intervention group demonstrated moderate chronic inflammation, with macrophages and lymphocytes indicating adipose tissue degeneration. The staining method used was hematoxylin and eosin (HE), and all photographs were taken at 20 × magnification field.

In the morphological analysis of adipose tissue with HE, they demonstrated that in the sample taken from the region that did not undergo treatment, that is, control, there was adipose tissue with normal characteristics, with adipocytes of standard size and absence of inflammation, since the sample collected from the region that received the treatment showed moderate chronic inflammation, with macrophages and lymphocytes indicating degeneration of the adipose tissue, as seen in Figure 3.

**FIGURE 4:**
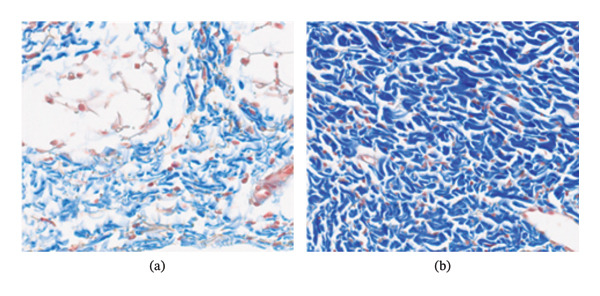
Photomicrographs of the intervention and placebo groups. (a) In the superficial dermis, the placebo group presented loose collagen tissue, with scattered fibers, and compatible with normal dermis. (b) The intervention group showed a large amount of collagen deposition with thick and organized fibers. The staining method used Masson’s Trichrome (MT), and all photographs were taken at 20 × magnification field.

In the morphological analysis of the tegument tissue with Masson’s Trichrome, the collagen fibers of the sample collected from the placebo were evaluated, which demonstrated loose collagen tissue, with scattered fibers and little cohesiveness, and compatible with normal dermis, whereas the sample collected from the intervention area showed large amount of collagen deposition with thick and organized fibers, as seen in Figure 4.

## 4. Discussion

The present research evaluated the possible physiological effects of tecar therapy on the tegument tissue and subcutaneous adipose tissue of women with fat located in the abdominal region. The results showed a possible promising effect of the resource, but it is necessary to understand and interpret each one of them and what their possible beneficial effect is in the conservative treatment of sagging skin and localized fat.

It is known that skin sagging is caused by physiological alterations in the epidermis, dermis and its appendages, and localized fat by hypertrophy and/or hyperplasia of adipocytes, both are multifactorial esthetic conditions that have been gaining more evidence in the search for treatments to improve of self‐esteem and quality of life. Thus, several resources have been developed for the noninvasive treatment of these conditions, one of which is known worldwide as RF [8–10].

The tecar therapy is a type of RF where heat will be produced by means of an electric current and not an electromagnetic field; however, we can say that its physiological effects evidenced in this study corroborate the similarity of the effects in the integumentary and adipose tissue of RF [11, 12]. The present study demonstrated that G‐1 presented a decrease in the thickness of the superficial subcutaneous adipose tissue in the supraumbilical and infraumbilical regions, both with *p* = 0.002 in the comparisons between the baseline and the end of the 10 treatment sessions and when comparing the G‐1 to G‐2 with *p* = 0.001. These data can be confirmed by the morphological analyzes of the adipose tissue, which demonstrated that in G‐1, there was a moderate chronic inflammation, with macrophages and lymphocytes indicating degeneration of the adipose tissue.

The degeneration of adipose tissue is known as lipolysis, which is a complex biological process, mediated by several molecular pathways stimulated endogenously and exogenously. In the case of tecar therapy, it stimulates lipid metabolism by heating tissue “heat” [13–15]. Vale et al. [13] performed a systematic review and meta‐analysis on the effects of heat as a RF resource on adipose tissue. After analyzing the articles, the authors revealed that RF can cause a significant increase *p* = 0.04 in the lipolysis process, proven by the increase in glycerol release and morphological changes in adipocytes that corroborate the findings in this study, indicating that the lipolysis process is caused by the increase in temperature [15, 16].

According to Kaplan and Gat, when temperatures within the 42°C range are applied, there is already lipolytic stimulation in the superficial adipose tissue. In addition, authors have revealed that the lipolysis process can be potentiated by raising the temperature, which stimulates the release of adrenaline and noradrenaline, at the same time, heating the tissue is directly related to the phenomenon of vasodilation, which generates an increase in the perfusion and oxygenation of the fabrics. Thus, oxidation and lipid turnover will be enhanced, culminating in a decrease in the volume of adipocyte cells (hypotrophy) [15–17].

The positive results in the ultrasound evaluations and histological analysis of the adipose tissue corroborate the evidence of body remodeling, seen in the present study in the results of the decrease in the umbilical and infraumbilical circumferences (*p* < 0.04 and *p* < 0.02) by the perimetry measurement. It should be noted that women have greater difficulty in reducing circumference measurements in specific regions of the body, such as thighs, abdomen, and buttocks, due to greater estrogen production with consequent increase in the concentration of alpha‐2‐adrenergic receptors in these places, making the lipolytic process more difficult. However, our study demonstrated that tecar therapy can be a beneficial and supportive resource in the treatment of body remodeling, since the thermal action of RF, in addition to promoting the reduction of adipocyte volume, acts to reduce circumference by acting on the thermal contraction of collagen fibers with induction of progressive neocollagenesis [16–19].

The present study also demonstrated beneficial results in relation to the integumentary tissue seen by the histological analysis; in G‐1, the dermis showed a greater amount of fibroblasts, inflammatory cells, and even newly formed blood vessels corroborating with neocollagenesis, which favors the improvement of the skin, with greater blood circulation, oxygenation and nutrients, and the consequent realignment of collagen fibers and even a reduction in body circumference [5–7, 20–22]. This effect on the integumentary tissue is possible due to two important factors, the peak temperature reached and the time during which heat is maintained, the first effect being triggered by collagen thermocontraction and the second related to stimulating fibroblasts to synthesize new collagen and elastin fibers [23, 24].

Brightman et al., [23] demonstrated the results in histological analyzes of samples of integumentary tissue from women submitted to RF treatment, the histological results showed an increase in cellular components of the extracellular matrix of the papillary dermis, that is, fibroblasts, together with an increase in composition of collagen fibers, which prove that controlled diathermy generates increased density in the papillary dermis and even reticular dermis, showing greater organization in relation to the orientation of collagen fibers [23].

Through the findings of this study, it is possible to verify that tecar therapy presents interesting clinical and physiological effects when in contact with the tissue, proving to be an effective and safe therapy in the treatment of esthetic conditions related to sagging skin and localized fat. However, due to study limitations, such as sample loss during reassessments, the authors suggest that new studies be carried out in order to better demonstrate and clarify the clinical effects found.

## 5. Conclusion

Tecar therapy proves to be a safe and effective resource in the treatment of esthetic conditions related to skin sagging and reduction of superficial adipose tissue, being an innovative technology capable of inducing biological stimuli that improve the appearance of the skin, resulting in body remodeling and tissue reduction adipose.

## Author Contributions

Graduate Degree Stephani de Almeida: physiotherapist, experimental application of the technique, and revision of the results. Graduate Degree Isabella Benatti Santiago: biomedicine, application of the clinical protocol, and statistical data. PhD Cintia Cristina Santi Martignago: physiotherapist, protocol preparation, and revisions. MSc. Michele Akemi Nishioka: physiotherapist, protocol preparation and revisions, and publication of the study report. PhD Patricia Brassolatti: physiotherapist, protocol preparation and revisions, and publication of the study report. MSc Jéssica Helena Franco Dorigatti: biomedical, protocol preparation, and revision. MSc Fabiele Chieregato: graduation in aesthetics, experimental application of the technique, and revision of the results. PhD Ana Carolina Araruna Alves: physiotherapist, protocol preparation and revisions, and publication of the study report. Stephany Luanna Queiroga Farias: physiotherapist, application of the clinical protocol, and statistical data. PhD. Patricia Froes Meyer: project orientation and revisions. B.A. José Ricardo de Souza: support with the development and production of equipment and study development.

## Funding

The present study does not have a funder since a partnership was established between the research groups involved.

## Conflicts of Interest

The authors declare no conflicts of interest.

## Data Availability

Data sharing is not applicable to this article as no datasets were generated or analyzed during the current study.
